# Optical coherence tomography angiography in multiple sclerosis: A cross-sectional study

**DOI:** 10.1371/journal.pone.0236090

**Published:** 2020-07-23

**Authors:** Roberta Farci, Arturo Carta, Eleonora Cocco, Jessica Frau, Maurizio Fossarello, Giacom Diaz

**Affiliations:** 1 Eye Clinic, University of Cagliari, Cagliari, Italy; 2 Department of Medicine and Surgery, Ophthalmology Unit, University of Parma, Parma, Italy; 3 Department of Medical Sciences and Public Health, Multiple Sclerosis Center Binaghi Hospital, University of Cagliari, Cagliari, Italy; 4 Biomedical Science Department, University of Cagliari, Cagliari, Italy; Weill Cornell Medicine-Qatar, QATAR

## Abstract

**Objectives:**

To evaluate retinal axonal density and retinal capillary flow density (CFD) variations in patients affected by multiple sclerosis (MS) as documented by Optical Coherence Tomography Angiography (OCT-A).

**Material and methods:**

A cross-sectional study was performed in a tertiary university eye hospital on 94 eyes from 48 MS patients compared to 37 eyes from 23 matched controls. MS patients were divided in two groups: those with previous episodes of optic neuritis (MS ON+, 71.4%) and those without any previous visual complaint (no optic neuritis group, MS ON, 28.6%). Patients underwent macular and optic nerve head OCT-A with Optovue XR Avanti (Optovue, Freemont, California) after that preliminary evaluation of the ganglion cell complex (GCC) and of the retinal nerve fiber layer (RNFL) was achieved for each single eye by SD-OCT. CFD was evaluated in three different retinal layers of MS patients and controls: superficial capillary plexus (SCP), deep capillary plexus (DCP) and the choriocapillaris layer (CL). Each layer was analyzed in 18 preset subregions automatically detected by the system. CFD values were then correlated to the RNFL thickness and GCC thickness in the groups: p values were computed by t-tests between each group of MS patients and controls. A p-value of <0.05 was considered significant.

**Results:**

A significant difference in the overall CFD values was found between ON+ and ON- patients when compared to controls in 18 subregions of SCP. Furthermore, a significant difference was found between MS patients and controls in 16 subregions analyzed corresponding to the CL layer without difference between the two MS subgroups (ON^+^ and ON^-^)

**Conclusions:**

OCT-A when performed at the optic nerve head level and at the macular region is characterized by a reduction of retinal perfusion in a significant portion of MS patients independently if they had a previous history of optic nerve inflammation or not.

## Introduction

Multiple sclerosis (MS) is considered to be an autoimmune, inflammatory disease affecting the central nervous system, and is responsible for plaque formation and destruction of the myelin sheath with consequential axonal degeneration [1,2,3]. Its precise etiology remains unknown, although both genetic and environmental factors may influence the susceptibility to develop MS.

The visual system is typically involved in MS patients; optic neuritis (ON) represents the most common ocular manifestation of MS, which may be observed during the clinical course of the disease [4,5] in 30%–70% of patients, and it is the first clinical manifestation of the disease in up to 25% of cases [6]. The high percentage of visual system involvement has been documented in post-mortem analyses, which have revealed that 94%–99% of patients with MS have axonal loss and degeneration in their optic nerves, independently of whether they have had a history of optic nerve inflammation [7,8].

Spectral domain optical coherence tomography (SD-OCT) is a noninvasive technique that provides μm axial resolution in cross-sectional retinal imaging, to quantitate reliably the ganglion cell axonal thickness at the level of the peripapillary retinal nerve fiber layer (RNFL) and the macular region. It is clinically accepted that optic nerve atrophy and thinning of the peripapillary RNFL are two typical findings of patients with MS, with or without a history of ON. RNFL thickness has been shown to decrease by 10–40 mm in the 3–6 months following an episode of acute ON [9,10]. and RNFL reduction may occur even in the absence of ON episodes, representing a biomarker of disease progression [11,12].

Although the results from past reports are often controversial regarding vascular and neuronal damage, the frequent association between MS and vascular diseases suggests that vascular changes might contribute to neuronal or degenerative dysfunction in patients with MS. Recently, by using optical coherence tomography angiography (OCT-A) in patients with MS, it has been shown that ON is associated with rarefaction of the superficial and deep retinal vessels [13]. OCT-A is a new, noninvasive imaging technique that employs motion contrast imaging of high resolution volumetric blood flow to generate angiographic images in a matter of seconds without the use of dye. The en-face images of OCT angiograms can be visualized from the internal limiting membrane to the choroid as well as individual vascular plexus and segments of the inner retina, outer retina, and choriocapillaris [14].

To provide further characterization of the retinal flow density in MS patients, we used OCT-A and spectral domain-optical coherence tomography (SD-OCT) to evaluate comparatively the axonal densities of the peripapillary RNFL and macular regions, as well as the retina and capillary flow density (CFD) in MS patients.

## Materials and methods

### Study population

This study was performed at the Eye Clinic Department, University of Cagliari. The research protocols were conducted in accordance with the tenets of the Declaration of Helsinki. At the time of the start of our study, October 2016, we had made a formal request to the local IRB of the University of Cagliari for ethical approval. The IRB Commitee gave a preliminary verbal approval of our study as this is a “NONINTERVENTIONAL COHORT RETROSPECTIVE STUDY” which completely adheres to the tenents of the declaration of Helsinki. Unfortunately, before the IRB gave a full written approval of the study. The Commitee was closed in March 2017 in favour of two different “Ethical Commitees” named Comitato Etico Indipendente della A.O.U. di Cagliari (C.E.I.) established on 5th September 2017. In the meantime we had no Ethical Commitee. All patient data were completely anonymized, de-identified and aggregated before access and analysis.

Authors take the full responsibility for any ethical request about this study. Written informed consent was obtained from each participant after an explanation of the nature of the study. Patients with MS were, in part, referred by the MS Centre of Cagliari, Ospedale Binaghi and, in part, by the Emergency Department at the Eye Clinic of San Giovanni di Dio, Cagliari from February 2016 to September 2016. OCT-A and SD-OCT data were obtained from 94 eyes of 48 patients with MS, and from 37 eyes of 2 controls. For CFD analysis, the eyes of MS patients were subdivided into two groups: eyes with previous episodes of ON (MS ON^+^, 71.4%) and eyes with a negative history of ON (MS ON^-^, 28.6%). Demographic data of patients and controls are summarized in Table 1. Characteristics of the eyes used for OCT-A and SD-OCT analyses are shown in Tables 2 and 3, respectively.

**Table 1:** Demographic data of MS patients and controls used in this study.

	Total	age (average ± SD)	
	Number	females	males
**MS cases**	48	41.7 ± 13.6	40.2 ± 12.5
**controls**	23	50.2 ± 13.9	54.0 ± 13.7

**Table 2:** Eyes submitted to OCT-A analysis.

	total	females	males	right eyes	left eyes	ON+	ON-
	number	(%)	(%)	(%)	(%)	(%)	(%)
**MS cases**	91	86.8	13.2	51.6	48.4	71.4	28.6
**controls**	29	34.5	65.5	55.2	44.8	-	-

**Table 3:** Eyes submitted to SD-OCT analysis.

	Total	females	males	right eyes	left eyes
	number	(%)	(%)	(%)	(%)
**MS cases**	94	84.0	16.0	50.6	49.4
**controls**	37	56.8	43.2	51.3	48.6

A medical history of ON prior to enrollment was determined by self-reports and physicians’ reports, and confirmed by record reviews. Patients with MS were also analyzed retrospectively with OCT-A for either optic nerve head (ONH) RNFL values or for ONH other than macula CFD. In addition, three eyes of three MS patients had acute ON. One patient had a vascular disease (systemic vasculitis). Forty patients were affected by relapsing-remitting MS, four by primary progressive MS, and four by clinically isolated syndrome. Other ophthalmic symptoms of patients previously objectified were visual field scotomas, diplopia, nystagmus, anisocoria, and a transitory decrease in visual acuity. A total of 70% of the patients received pharmacological treatment for MS (Azatioprin, Tecfidera, R44, Rebif, Plegredy, Gilenya, beta interferon, Aubagio, and Copaxone).

The inclusion criteria were MS diagnosis (any subtype: relapsing-remitting, primary progressive, and secondary progressive), with or without previous ON, and intraocular pressures < 21 mmHg in both eyes. Patients with macular degeneration, glaucoma, diabetic retinopathy, or vitreomacular diseases were excluded.

### Analysis

The CFD of the macular region and peri-papillary radial plexus, as well as the RNFL and ganglion cell complex (GCC), were evaluated with OCT-A (Optovue XR Avanti Device, Fremont, CA, USA). The scan had an A-scan rate of 70,000 scans/s, using a light source centred on 840 nm. OCT-A identifies red blood cell movement by means of a decorrelation (i.e. negative correlation) signal [15,16]. between consecutive scans over 6 × 6 mm subregions; the brighter the vessel appears, the faster the flow [16]. The efficiency of detecting the flow signal has been improved by use of the split-spectrum, amplitude-decorrelation angiography algorithm (SSADA) [17], which divides the OCT spectrum into narrower spectral bands and averages the decorrelation of these bands. This process significantly improves the signal to noise ratio without extending the acquisition time [18]. Motion artifacts are removed by aligning the images in x and y directions, and then by merging the image [19]. En-face projection of the flow signal internal to the retinal pigment epithelium creates the retinal angiograms. Two scans were performed for each eye, and the scan with better signal strength was chosen for analysis. Images with a signal strength index below 50 or significant residual motion artifacts were excluded from the study.

The flow density map software (AngioAnalytics, version 2015.100.0.35), an OCT-A tool, was used to measure CFD as the percentage of the sample area occupied by vessel lumens, after intensity thresholding and segmentation of images.

**Fig 1:**
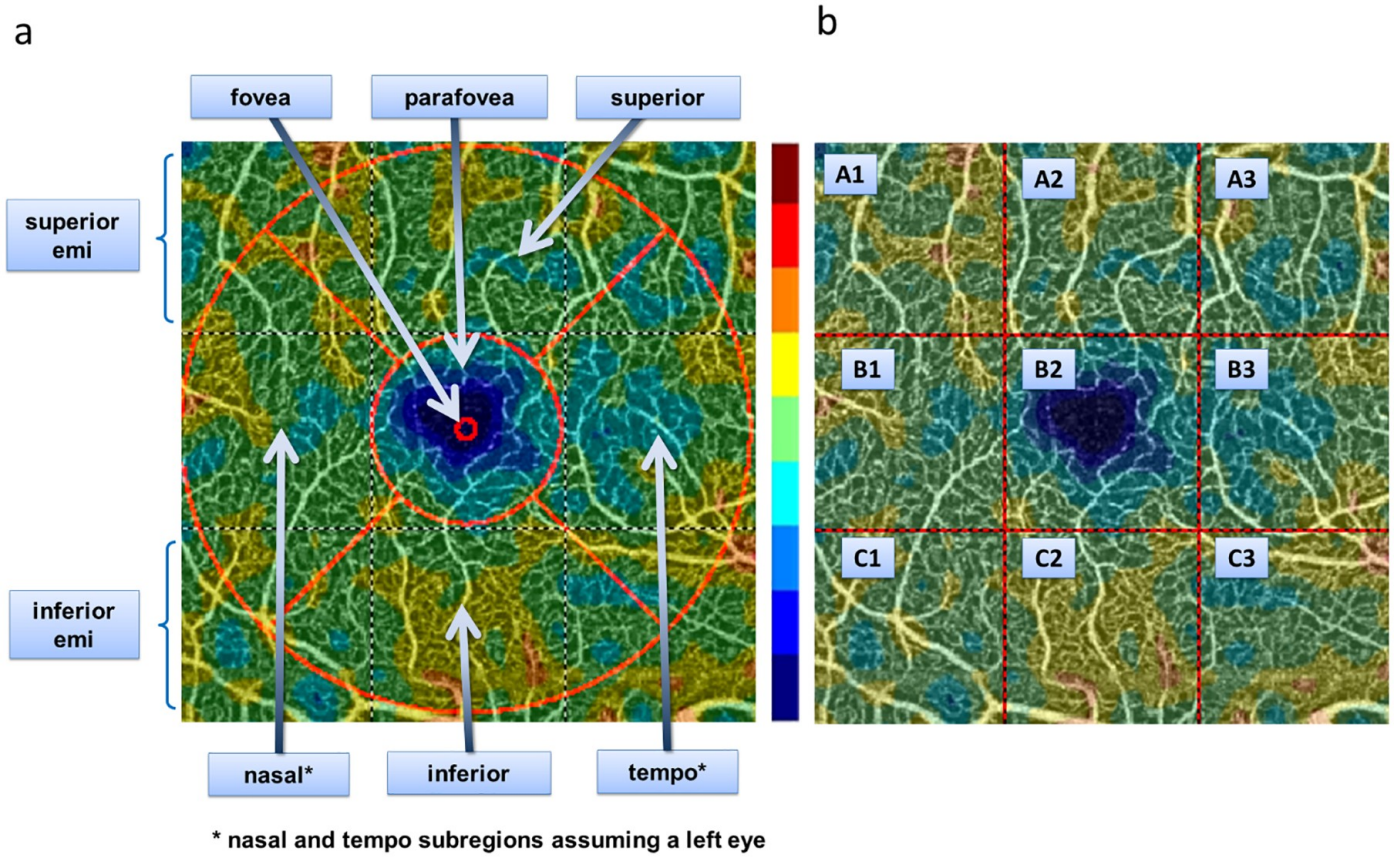
Measurements of OCT-A images. Two sets of OCT-A measurements were obtained: one from (A) 9 preset macular regions and one from (B) 9 grid-based regions. The range of colors, from blue to brown, represent the capillary flow density (blue = low, brown = high).

The CFD was evaluated in three different retinal layers: choriocapillaris layer (CL), deep capillary plexus (DCP) and superficial capillary plexus (SCP) [15] and, within each layer, in nine preset subregions automatically detected by the system (denoted as whole fovea, fovea, parafovea, superior hemi, inferior hemi, temporal, superior, nasal, and inferior; Fig 1A) and in nine subregions identified by a 3 × 3 square grid and (denoted as A1, A2, A3, B1, B2, B3, C1, C2, and C3; Fig 1B).

SCP and DCP were automatically segmented, whereas the CL was manually segmented. The CFD box was manually drafted from the macular region to the ONH localization. The thickness of the same retinal subregions, with the exclusion of the whole fovea, was also assessed. In addition we measured the thickness of GCC and RNFL of controls and MS patients. Owing to the unbalanced number of males and females and the different average age of the groups of subjects, statistical comparisons (controls vs. MS NO^+^, controls vs. MS NO^-^, controls vs. all MS, and MS NO^+^ vs. MS NO^-^) were made following ANCOVA adjustment for age and gender covariates, using the R package ‘rstatix’. The resulting p values were finally verified by the Benjamini-Hochberg method to keep a cumulative false discovery ratio among all test, among each retinal layer, less that 5%.

## Results

### 1. Retinal layer CFD as determined by OCT-A

**Fig 2:**
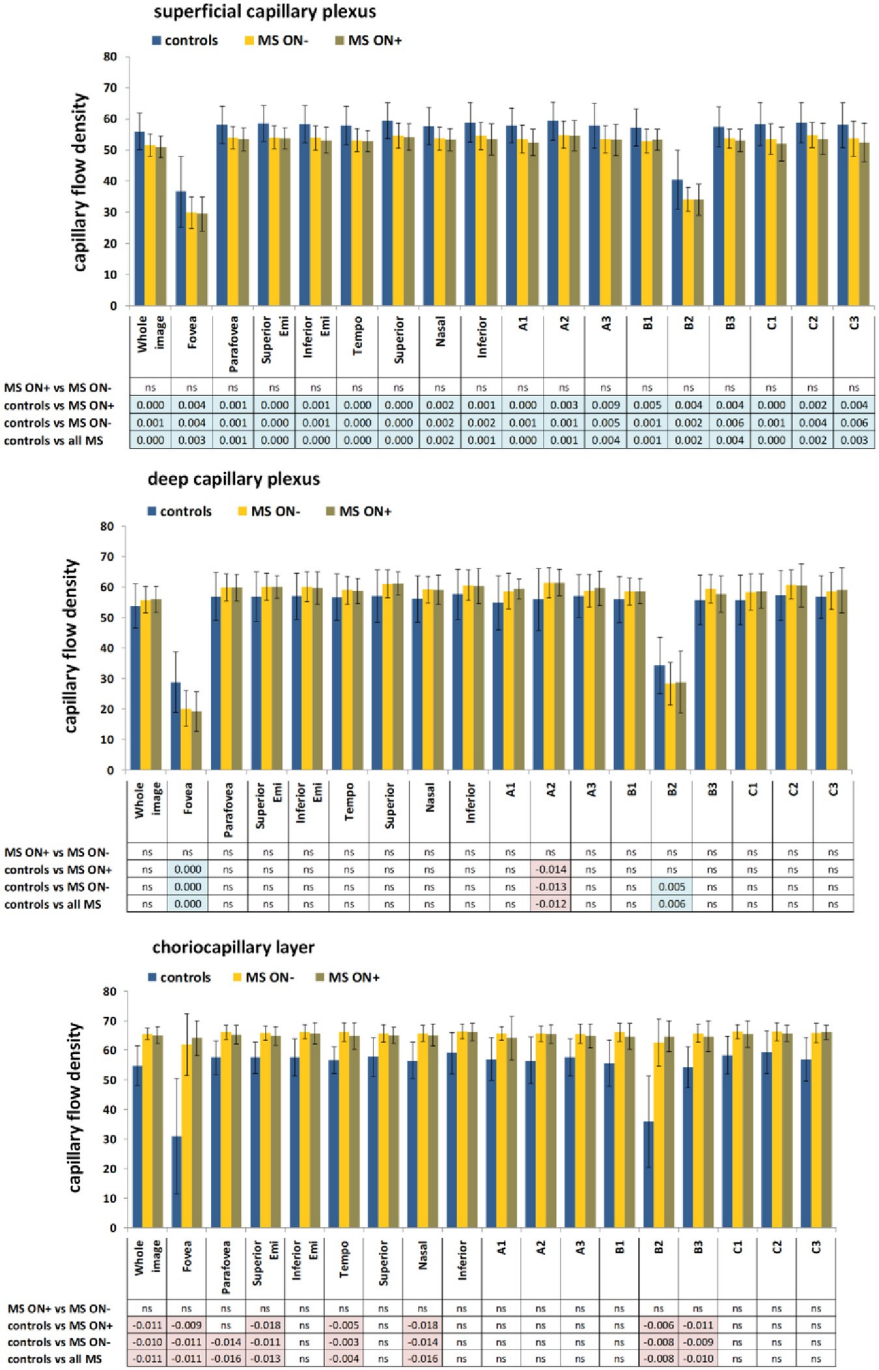
OCT-A CFD measurements in MS patients and controls. CFD measurements were made in the (A) choriocapillary layer, (B) deep capillary plexus and (C) superficial capillary plexus. Plots show the measurements of the 9 preset regions (whole image, fovea, fovea, parafovea, superior hemi, inferior hemi, temporal, superior, nasal, inferior) and 9 regions defined by a 3 x 3 square grid (A1, A2, A3, B1, B2, B3, C1, C2, C3). Bars indicate means and standard deviations. Comparisons were made following ANCOVA adjustment for age and gender covariates. P values were also verified by the Benjamini-Hochberg method to keep a cumulative false discovery ratio among all test less that 5%. Significant p values are shown in conventional colors to denote the direction of the change: cyan or orange p values mean that the average of the first group is significantly higher or lower, respectively, than the average of the second group.

No significant differences were found between ON^+^ and ON^-^ MS patients, in any region of the four retinal layers, either by comparing paired groups (between eyes with or without ON of the same patients), or by comparing unpaired groups (between eyes with or without ON independently of the patient). Significant differences between MS patients and controls were found in CFD values of all 18 subregions of the CL (p max 0.001) and all 18 subregions of the SCP (p max 0.003), but only in two subregions of the DPC, fovea and B2, which are partly superimposable (p max 0.001). Data are shown in Fig 2.

### 2. Retinal layer thickness as measured by OCT-A

**Fig 3:**
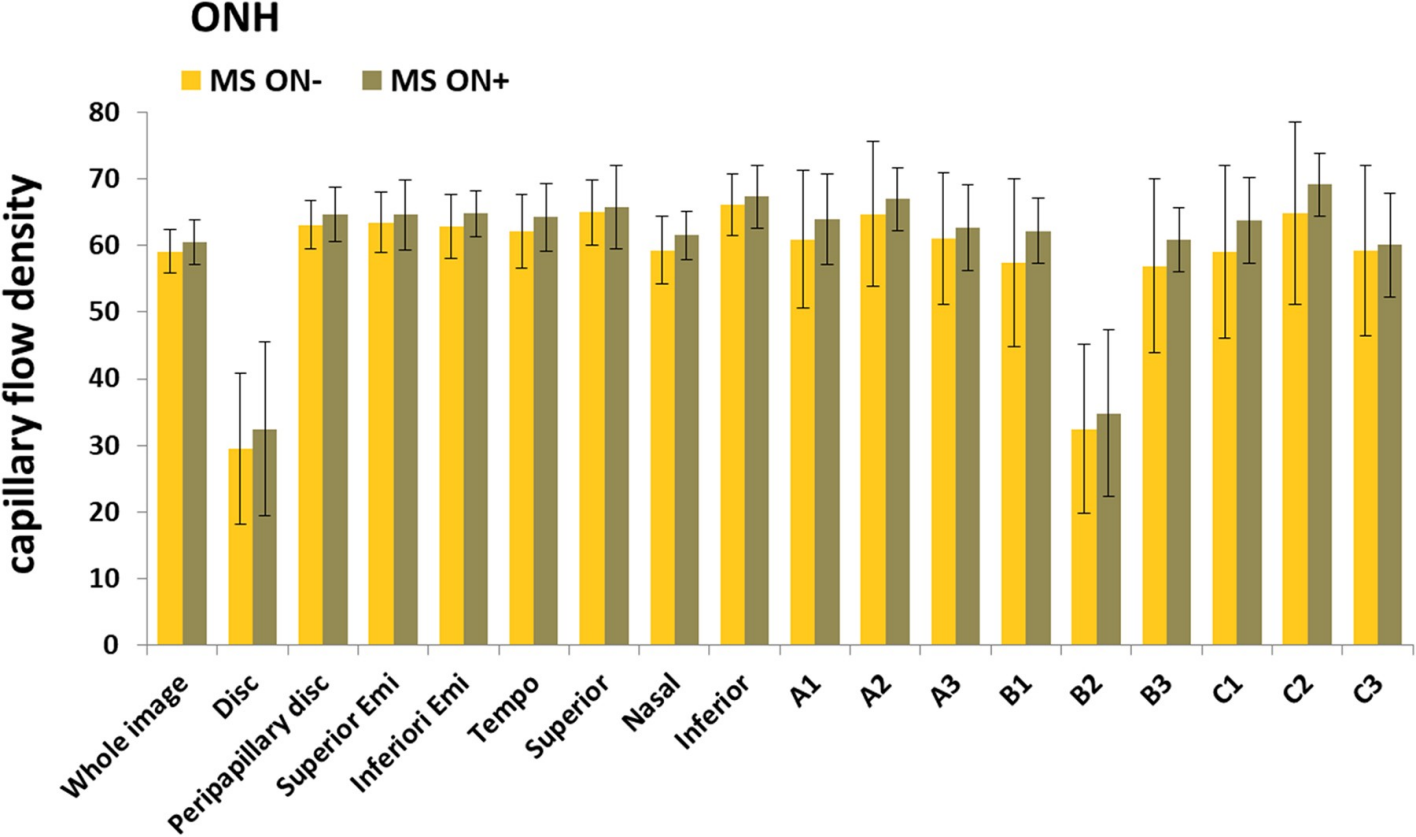
OCT-A thickness measurements in MS patients and controls. CFD measurements were made in the (A) choriocapillary layer, (B) deep capillary plexus and (C) superficial capillary plexus. Plots Fig 3. OCT-A measurements of the thickness of (A) choriocapillary layer, (B) deep capillary plexus and (C) superficial capillary plexus, in MS patients and controls. Statistical analysis and conventional p values colors are the same as those described in Fig 2.

Unlike CFD, the thicknesses of almost all subregions of all retinal layers exhibited significant changes between MS ON^+^ and MS ON^-^ patients in almost all preset subregions, except that in the fovea and nasal (p max 0.025). On the other hand, the comparison between controls and MS patients showed different data in the three retinal layers, with a relatively high number of significant changes in the SCP (p max 0.035), followed by the DCP (p max 0.031), and no changes in the CL. However, when statistically significant, all changes denoted a progressive decrease of thickness from controls to MS ON^-^, and from MS ON^-^ to MS ON^+^, in all retinal layers. Data are shown in Fig 3.

### 3. GCC as measured by SD-OCT

**Fig 4:**
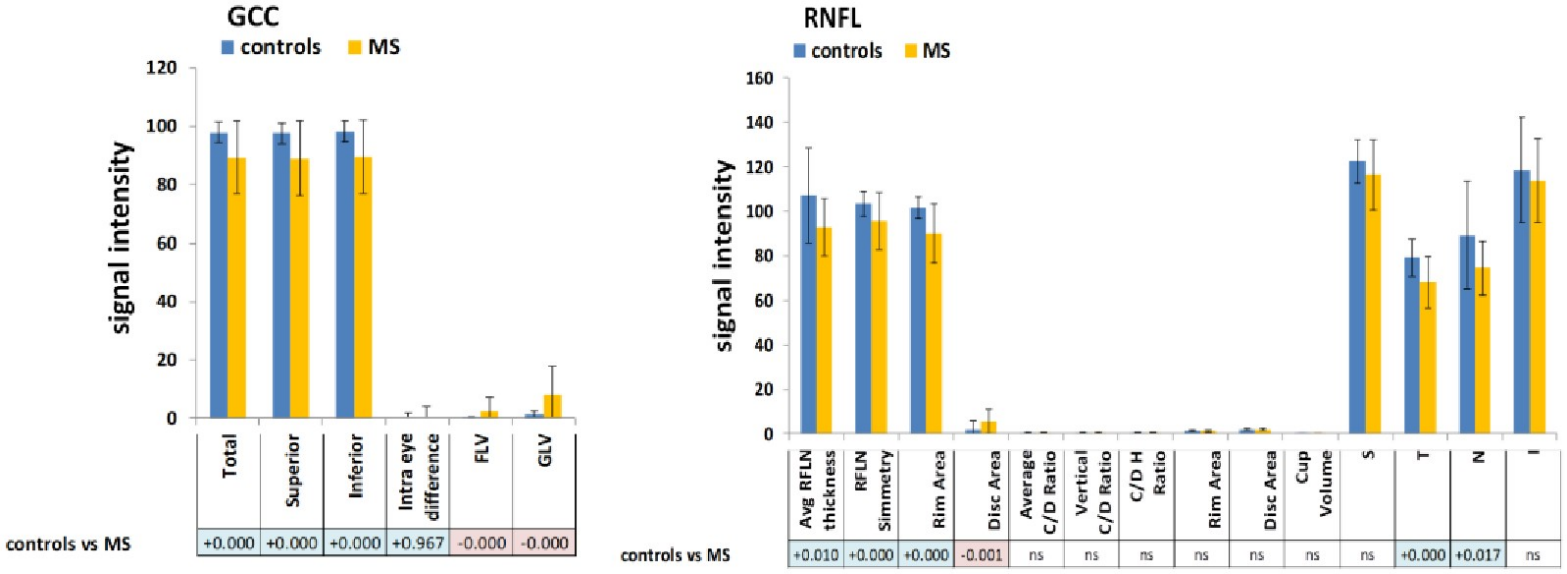
GCC and RNFL measurements by SD-OCT. (A) GCC signal in SD-OCT in MS patients and controls. (B) RNFL signal in SD-OCT in MS patients and controls. Statistical analysis and conventional p values colors are the same as those described in Fig 2.

A significant decrease of GCC thickness was found in MS patients compared to controls in the total, superior, and inferior subregions (p max 0.004). On the other hand, MS patients exhibited a significant increase of focal loss volume (FLV) and global loss volume (GLV) (p max 0.033). No significant intra-eye changes were found. Data are shown in Fig 4A.

### 4. RNFL as determined by SD-OCT

A significant decrease of the RNFL thickness was found in five subregions (total thickness, symmetry, rim area, S, and T) of MS patients (p max 0.013). However, MS patients showed a significant increase of the disc area (p = 0.002). Data are shown in Fig 4B.

## Discussion

In the present study, we could observe by means of OCT-A performed at the optic nerve head level and at the macular region a reduction of capillary perfusion in a significant portion of MS patients, independently if they had a previous history of optic nerve inflammation or not. In particular, we have demonstrated for the first time a significant difference in CFD at all levels of retinal and optic disc tissues in MS patients. This reduction was highly significant in the correlations of the superficial capillary plexus in all 18 subregions and in the correlations of the deep capillary plexus with five subregions, which were not related to a previous episode of ON. Wang et al. [20] described a significant decrease in CFD regarding OCT-A of ONH, which was higher in patients with antecedent ON, assigning this finding to previous ONH inflammation and the consequential damage of the GCC; although, in their study, the pathogenetic hypothesis was not confirmed.

An analogous condition of ischemia has been described at the cerebral level by several authors who studied the vascular aspects of MS.

Rindfleisch [21] and Charcot [22] were the first to expose a theory involving a vascular role in the development of demyelinating lesions. Caprio et al. [23] In a recent review observe that the high degree of comorbidity between vascular disease and MS supports the hypothesis that vascular pathology may be an important factor causing neuronal dysfunction or degeneration. Also other authors advocate that vascular components may be initiating triggers for neuronal pathology and subsequent neurological manifestations of the disease [24–31].

Significant alterations in vascular architecture of plaques have been described, involving low expression of endothelin-1 [32] which is a vasoconstrictor peptide, regulated by a large number of vasoactive agents, endothelial cell adhesion molecules (ICAms, VCAMs, JAMs, and lymphoid chemokines), inflammatory mediators (TNF-α and IFN-ϒ), and platelet alterations resulting in vasospasm and reducing the cerebral blood flow; with endothelial junctional disorganization, pathological deoxy-hemoglobin deposits, immune cell extravasation, and metabolic disorders, culminating in the loss of neural and then glial cells [33–37]. According to a pathogenetic hypothesis of these authors, we have demonstrated for the first time a significant difference in CFD at all levels of retinal and optic disc tissues in MS patients. This reduction was highly significant in the correlations of the superficial capillary plexus in all 18 subregions and in the correlations of the deep capillary plexus with five subregions, which were not related to a previous episode of ON.

Ganglion cells and their axons, besides being the main retinal component around the optic nerve (90% of the retinal thickness), are also representative in the correlation of the macula (30%–35%). This reduction corresponds to brain atrophy (which was associated with general disability in MS patients), which is a cause of disability in MS [38].

The significant difference found between patients and controls regarding macular CFD is important because the reduction of the macular CFD is a diagnostic tool for MS patients and because we found a significant difference between controls and MS patients. We therefore propose that macular CFD represents a new parameter for the prediction of MS progression.

The depletion of ganglion cells was also confirmed by significant differences of thicknesses at all levels. A reduction of ganglion cell number contributes to the reduction of retinal volume correlating with the superficial and deep capillary layers. Our results regarding choroidal thickness confirmed the results of Esen et al. and Garcia-Martin et al. [39,40], which suggest a potential role for vascular dysregulation in the pathophysiology of MS. We suggest that a reduction of GCC and, consequently, of the retinal tissue, implies a loss of vascularization at this level. We propose that the reduction of retinal tissue, including the loss of GCC and RNFL found by SD-OCT, leads to hypoxia. This was confirmed by the findings of microcystic edema and thickening of the inner nuclear layer (INL), which seems to share an ischemic pathogenesis [41].

The absence of a difference between MSON^-^ and MSON^+^ patients indicated that ganglion cell depletion in the macular area was directly related to the activity of MS, and was independent of a previous history of optic nerve inflammation.

We found a significant difference regarding RNFL data between patients and controls. The RNFL layer represents the innermost layer of the retina and it is formed by retinal ganglion cell axons, which are myelinated only after they pass through the lamina cribrosa. The mechanism of atrophy of the RFNL following visual pathway lesions in MS is incompletely understood. It has been postulated that it could result from direct loss of the cellular body (RGC), from retrograde degeneration of axons following their loss in the optic nerve, or from the subsequent atrophy of the proximal optic nerve axons in the RFNL following focal optic nerve axon loss [42]. Moreover, retrograde trans-synaptic (trans-geniculate) degeneration of the GCC due to MS lesions within the posterior optic pathways could cause RNFL loss even in absence of ON, even if it is less evident compared with those observed following ON [43,2]. It has been reported that acquired unilateral occipital damage causes a thinning of the RNFL and optic tract, confirming the existence of retrograde trans-synaptic degeneration of neurons in the human visual pathway [44,45,46,47]. However, MS patients who had previously suffered from ON exhibited more intense atrophy in the visual cortex, suggesting that the damage cascade may have also proceeded in the anterograde direction [45,48].

However, no significant correlation between OCT analyses of the CFD and GCC was found in our patients, suggesting the maintenance of a sufficient control of blood flow in the middle retinal layer, regardless of innermost retinal layer ischemia, as postulated by Wang [20].

It is worth mentioning that the blood-brain barrier seems to break down due to the systemic hypercoagulability status in MS patients. In a similar manner, we believe that an analogous inflammatory process may occur in the blood-retinal barrier. A primary network of microglia is localized at the INL level, which acts as a retinal barrier, and is susceptible to fluid accumulation during inflammation processes. Approximately 20% of MS patients show retinal periphlebitis and 50% of the patients exhibit KIR4 [49] antibodies against Mueller cells. This receptor regulates potassium channels as a critical element for signal transmission from the retina to the brain. Therefore, these antibodies may play a role in the pathogenesis of retinal ischemia in MS patients.

This study presents several limitations: 1) measurements of ONH CFD have been taken only in the cases but not in the controls. 2) CFD measurements at different times, so that we cannot affirm that CFD are subject to change in different stages of the disease. 3) Pathogenetical hypothesis of CFD reduction in MS patients are still uncertain, so it does not allow us to define CFD as a biomarker of multiple sclerosis. In conclusion, we think that OCT-A is a promising technique to predict the disability of MS patients. OCT-A was able to detect reduction of macular and ONH perfusions in a significant percentage of MS patients, with or without a history of ON. OCT-A with the SSADA algorithm is highly repeatable and reproducible for CFD measurements of the ONH and macular subregions. However, further studies are necessary to validate this novel technology for the early detection and follow-up of MS patients.

## Supporting information

S1 Data (XLSX)

## Abbreviations

CFD: capillary flow density
DCP: deep capillary plexus
FLV: focal loss volume
GCC: ganglion cell complex
GLV: global loss volume
OCT-A: optical coherence tomography angiography
ON: optical neuritis
ON^+^, ON^-^: patients with or without optical neuritis
ONH: optic nerve head
RNFL: retinal nerve fiber layer
RPC: radial peripapillary capillary
SCP: superficial capillary plexus
SD-OCT: spectral domain optical coherence tomography
